# Raoultella planticola Infection in Urine

**DOI:** 10.7759/cureus.17985

**Published:** 2021-09-15

**Authors:** Joana Cohen, Mohsin Altaf, Muhammad Mushtaq, Dustin Stanley

**Affiliations:** 1 Family Medicine, Unity Health, Searcy, USA; 2 Internal Medicine, Unity Health, Searcy, USA; 3 Medicine, Arkansas College of Osteopathic Medicine, Fort Smith, USA

**Keywords:** raoultella planticola, uti, acute cystitis, klebsiella, histamine

## Abstract

*Raoultella planticola* is a gram-negative, aerobic, nonmotile bacteria that can be found in soil and water. This is a relatively rare organism with few case reports on it and only three reports of *R. planticola*-induced urinary tract infection (UTI) have been reported. Here we present a case of acute cystitis caused by *R. planticola* in a woman with atrial fibrillation and recurrent UTIs.

## Introduction

*Raoultella planticola* is a gram-negative, aerobic, nonmotile bacteria that can be found in soil and water [1]. It was formerly known as *Klebsiella planticola* but was later reclassified in the new genus of *Raoultella *in 2001 partly due to its histamine-producing properties, which *Klebsiella *does not have [2]. There has been one confirmed case report of *R. planticola*-caused urinary tract infection (UTI) in a pediatric patient, and three reports in adults [3-5].

## Case presentation

A 92-year-old woman with the past medical history of hypertension, paroxysmal atrial fibrillation on anticoagulation with Coumadin, recurrent UTIs, breast cancer with left mastectomy, and diverticulosis presented to our emergency department after being found by her neighbor on the floor with right lower leg bleeding. The patient stated she tripped over a wooden trash can. In the emergency department, she was found to be hypotensive with lacerations to her right lower extremity and left forearm. A complete workup was performed which revealed an international normalized ratio (INR) 1.5, hemoglobin of 8.2, a head CT without contrast that revealed no intracranial abnormality, and extremity X-rays that revealed no fractures.

The patient was then transferred to the care of the ICU for hemorrhagic shock. She was placed on Levophed for pressure support and received 2 units packed red blood cells, 1 unit fresh frozen plasma, and 10 milligrams vitamin K. She was started on vancomycin and cefepime for an elevated white blood cell count. Blood culture was found to be negative. Urinalysis revealed sterile pyuria. The urine was cultured and based on its growth pattern was determined to be *R. planticola. *This was resistant to ampicillin but otherwise antibiotic sensitive and the patient was deescalated to Rocephin. The patient was successfully weaned off Levophed and transferred to the general medical floor in stable condition.

Her UTI resolved after completing her course of Rocephin. Her Coumadin was discontinued as her bleeding risk far outweighed the benefit of thrombo-embolism prophylaxis in the setting of atrial fibrillation. The patient was noticed to have functional deficits in all areas of her activity of daily livings (ADLs), and she was transferred to acute inpatient rehab.

## Discussion

*R. planticola* is an aerobic, gram-negative rod predominantly found in water and soil. Case reports reveal* R. Planticola* has also been associated with acute cholecystitis, UTIs, pneumonia (PNA), bacteremia, necrotizing fasciitis, soft tissue infections, and scombroid poisoning [3-7]. It has also notably been isolated in human urine, stool, and sputum. The first reported infection was in 1986, but there has been an increasing number of associated reports since the year 2000 [8].

Given the scant amount of literature surrounding *R. Planticola*, its mechanism and pathogenesis remain uncertain. There is a possibility that its histamine-producing properties may be related to its pathogenesis [2]. At this time, it is not known if the presence of* *alcohol dehydrogenase plays a role in its ability to cause disease. Further research into how humans contract *R. Planticola *and its pathogenesis is warranted.

## Conclusions

In conclusion, *R. planticola* infection appears to be increasing, whether that is due to a better ability to distinguish it from *Klebsiella*, or that its prevalence is increasing is hard to say. However, it does appear to have a predilection for the immunocompromised and patients with recurrent UTIs. It should be considered on the differential for etiologies of UTI in that setting. Fortunately, to date, *R. planticola* is susceptible to most antibiotics with gram-negative coverage.
